# Infection-associated type IV secretion systems of *Bartonella* and their diverse roles in host cell interaction

**DOI:** 10.1111/j.1462-5822.2008.01171.x

**Published:** 2008-06-19

**Authors:** Christoph Dehio

**Affiliations:** Focal Area Infection Biology, Biozentrum of the University of BaselKlingelbergstrasse 70, CH-4056 Basel, Switzerland.

## Abstract

Type IV secretion systems (T4SSs) are transporters of Gram-negative bacteria that mediate interbacterial DNA transfer, and translocation of virulence factors into eukaryotic host cells. The α-proteobacterial genus *Bartonella* comprises arthropod-borne pathogens that colonize endothelial cells and erythrocytes of their mammalian reservoir hosts, thereby causing long-lasting intraerythrocytic infections. The deadly human pathogen *Bartonella bacilliformis* holds an isolated position in the *Bartonella* phylogeny as a sole representative of an ancestral lineage. All other species evolved in a separate ‘modern’ lineage by radial speciation and represent highly host-adapted pathogens of limited virulence potential. Unlike *B. bacilliformis*, the species of the modern lineage encode at least one of the closely related T4SSs, VirB/VirD4 or Vbh. These VirB-like T4SSs represent major host adaptability factors that contributed to the remarkable evolutionary success of the modern lineage. At the molecular level, the VirB/VirD4 T4SS was shown to translocate several effector proteins into endothelial cells that subvert cellular functions critical for establishing chronic infection. A third T4SS, Trw, is present in a sub-branch of the modern lineage. Trw does not translocate any known effectors, but produces multiple variant pilus subunits critically involved in the invasion of erythrocytes. The T4SSs laterally acquired by the bartonellae have thus adopted highly diverse functions during infection, highlighting their versatility as pathogenicity factors.

## Introduction

Type IV secretion systems (T4SSs) are supramolecular protein assemblies that mediate the intercellular transfer of protein or DNA substrates from a bacterial donor cell into various types of target cells, e.g. the transfer of DNA into recipient bacteria by bacterial conjugation, and the inter-kingdom transfer of bacterial effector proteins into the cytosol of eukaryotic host cells. Bacterial conjugation is a significant medical concern because it represents a mechanism for the transmission of virulence genes, antibiotic resistance genes and other fitness traits of pathogenic bacteria within bacterial populations (Ochman *et al*., 2000). Moreover, numerous pathogens have adopted T4SSs for the delivery of bacterial effector proteins into the cytoplasm of mammalian host cells in order to subvert host cellular functions. T4SSs represent crucial pathogenicity factors for important human pathogens such as *Helicobacter pylori*, *Legionella pneumophila*, *Bordetella pertussis*, *Brucella melitensis* and *Bartonella henselae* (Christie *et al*., 2005).

Type IV secretion systems consist of a substrate translocation channel spanning the two membranes of Gram-negative bacteria, and a surface filament extending from the bacterial envelope. The latter pilus structure is thought to establish initial contact with target cells. Subsequently, the translocation channel is believed to extend to the target cell membrane to facilitate substrate translocation (Christie *et al*., 2005). Using the nomenclature for the canonical VirB/VirD4 T-DNA transfer system of the tumorigenic plant pathogen *Agrobacterium tumefaciens*, T4SSs are macromolecular assemblies of at least 10 essential components termed VirB2–VirB11, and an associated substrate recognition receptor known as the type IV secretion coupling protein (T4CP) VirD4. Several basic molecular features of T4SSs are known, including atomic structures for the T4CP and six out of the 10 essential T4SS components, protein–protein interaction maps of the transporter constituents, translocated substrates and substrate recognition sequences (Christie *et al*., 2005). In addition, the sequential interactions of a DNA substrate with individual components of a T4SS have been mapped during intercellular substrate translocation (Cascales and Christie, 2004). However, in contrast to the successful visualization of the injectisome complexes of type III secretion systems (T3SSs), which also mediate inter-kingdom protein trafficking (Marlovits *et al*., 2004), no high-resolution images of the macromolecular assembly of a T4SS have been reported.

Bartonellae are mammalian pathogens that infect erythrocytes and vascular endothelial cells as major target cells (Dehio, 2004). Transmission between mammalian hosts occurs via blood-sucking arthropods or via direct contact. Bartonellae are host-restricted pathogens and the establishment of a long-lasting intraerythrocytic bacteraemia as hallmark of erythrocyte infection takes place exclusively in one or a few reservoir host(s). In contrast, endothelial cells appear to represent a major target cell type for bacterial colonization in the reservoir host(s) and probably also in incidentally infected hosts (Dehio, 2005). Upon transmission to a reservoir host the infection cycle is initiated by the colonization of the so-called primary niche, which is considered to include the vascular endothelium as major cellular constituent, but possibly also cells of the reticuloendothelial system. Approximately on day five of infection, bacteria get synchronously seeded from the primary niche into the bloodstream, where they efficiently infect erythrocytes, but also reinfect the primary niche. The latter event leads to synchronous releases of bacteria into the bloodstream in approximately 5-day intervals until an antibody-dependent immune response terminates the cyclic course of infection (Koesling *et al*., 2001; Schulein *et al*., 2001). The infection of erythrocytes involves firm bacterial adhesion, followed by bacterial internalization into a membrane-bound compartment in which bacteria replicate. However, bacterial growth is ceased after few divisions, and intraerythrocytic bacteria circulate in the bloodstream for the remaining lifespan of the infected erythrocyte, which can last for several weeks to month (with the exception of *Bartonella bacilliformis*, which typically causes lysis of infected erythrocytes). The resulting long-lasting intraerythrocytic infection represents a specific adaptation to the mode of transmission by blood-sucking arthropods (Schulein *et al*., 2001).

**Fig. 1:**
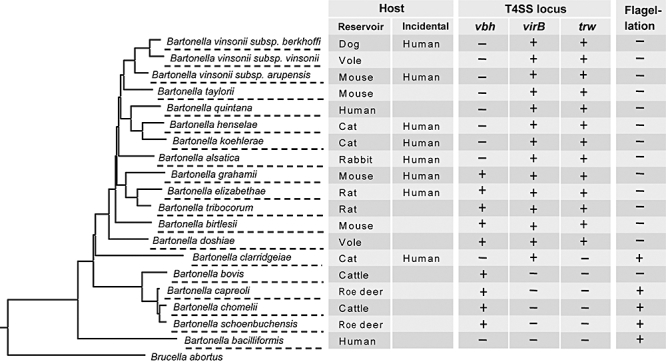
Distribution of T4SSs and flagella in the genus *Bartonella*. Left: Phylogenetic tree of the genus *Bartonella* based on multilocus sequence analysis (MLSA, Saenz *et al*., 2007). Right: Summary table of the host specificity and the presence and absence of loci encoding T4SSs and flagella in the different *Bartonella* species. Based on the lack of sufficient DNA sequence information for MLSA, the species *Bartonella washoensis* (ground squirrel-specific, zoonotic), *Bartonella peromysci* (deer-/mouse-specific), *Bartonella phoceensis* (rat-specific), *Bartonella rattimassiliensis* (rat-specific), *Bartonella talpae* (mole-specific) (Dehio, 2005), *Bartonella rochalimae* (Eremeeva *et al*., 2007) and *Bartonella tamii* (Kosoy *et al*., 2008) are not included in the phylogenetic tree and further analysis.

Bartonellae may cause a wide range of clinical manifestations, ranging from asymptomatic infection to fatal disease – depending on both the infecting species/strain and the immunological status of the host (Dehio, 2005). *B. bacilliformis* is a deadly human-specific pathogen that causes Carrión's disease in endemic areas of the Andes. The acute phase, called Oroya fever, is characterized by an intraerythroctic bacteraemia that results in haemolytic anaemia with a fatality rate of up to 85% if untreated. A subsequent chronic phase, known as verruga peruana, manifests in vascular tumours that result from the massive proliferation of colonized endothelial cells (Dehio, 2005). In contrast to the highly virulent *B. bacilliformis*, all other bartonellae commonly cause infections of low virulence potential in their specific mammalian reservoirs. For example, the human-specific pathogen *Bartonella quintana* causes trench fever, a rather mild cycling fever with a periodicity of 5 days. More severe disease manifestations, such as bacillary angiomatosis (characterized by the formation vascular tumours similar to the verruga peruana), may result in a state of immunosuppression. Another example is the zoonotic pathogen *B. henselae* that causes asymptomatic infections in its feline reservoir. However, incidental infection of humans by cat scratch or bite can result in disease, such as cat scratch disease (a self-limiting infection of lymph nodes) in immunocompetent, or bacillary angiomatosis in immunocompromised patients. Further to *B. henselae*, eight other *Bartonella* species are considered as zoonotic agents that cause asymptomatic infections in their respective animal reservoirs. Finally, the remaining 13 *Bartonella* species that have not been associated with human infection typically cause as well asymptomatic infections in their respective mammalian reservoirs, i.e. *Bartonella tribocorum* infecting the rat as reservoir host (Heller *et al*., 1998). Phylogenetic analysis of the genus *Bartonella* indicated an isolated position of the deadly pathogen *B. bacilliformis* as sole representative of a deep-branching ancestral lineage, whereas all other bartonellae evolved more recently in a lineage that radiates as a result of their adaptation to different mammalian reservoirs (Fig. 1) (Saenz *et al*., 2007).

Since 1993, the genus *Bartonella* has expanded from one species, the founding member *B. bacilliformis*, to currently 24 species. In the same period the establishment of bacterial genetics, as well as animal and cell culture infection models facilitated a significant progress in our understanding of the molecular and cellular basis of *Bartonella* pathogenesis. Three distinct T4SSs, VirB/VirD4, Vbh/TraG and Trw, as well as a set of T4SS-translocated effector proteins, have now been identified as *bona fide* pathogenicity factors of the bartonellae. This review outlines the adaptive evolution of these secretion systems, their roles in *Bartonella* infection and their molecular properties in subverting host cell functions.

## The VirB/VirD4 T4SS and its translocated effectors mediate subversion of endothelial cell functions

**Fig. 2:**
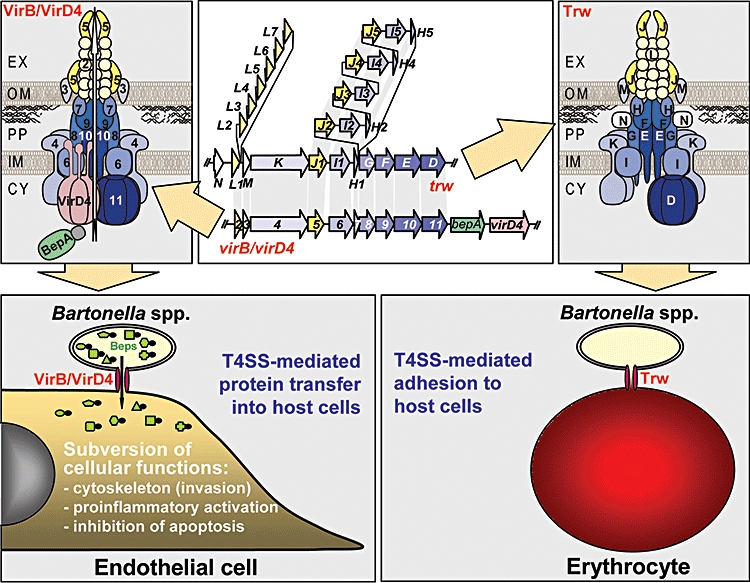
Genetic organization and model of the architecture of the VirB/VirD4 and Trw T4SS, and their role in host cell infection. Top: Genetic organization of the T4SS loci *virB/virD4* and *trw* (middle) and hypothetical model of the architectures of the VirB/VirD4 (left) and Trw (right) systems. The VirB/VirD4 system constitutes a complete T4SS with the inner membrane-localized ATPases VirD4 (T4CP), VirB4 and VirB11 that energize the secretion process, the components VirB3, VirB6, VirB8, VirB9 and VirB10 considered to build a secretion channel across the inner membrane, periplasm and outer membrane, and the pilus-associated components VirB2 (pilin) and VirB5 (minor pilus-associated component). Substrates such as the effector protein BepA are considered to interact via their C-terminal translocation signal with the T4CP VirD4 prior to secretion by the VirB T4SS. The Trw system of *Bartonella* – other than the closely related Trw conjugation system of plasmid R388 – lacks a T4CP (TrwB in R388) and does not secrete any known substrate. Due to tandem gene duplications the *Bartonella* Trw system expresses multiple variant copies of the pilus-associated components TrwL (pilin) and TrwJ (minor pilus-associated component), EX, extracellular matrix; OM, outer membrane; PP, periplasm; IM, inner membrane; CY, cytoplasm; Bottom: Roles of these T4SSs in host cell infection. The VirB/VirD4 system (left) injects a cocktail of Bep effectors into endothelial cells and thereby mediates the subversion of multiple host cells functions. The Trw system (right) expressing variant pilus components is considered to play a critical role in the adhesion to and invasion into erythrocytes.

The first T4SS in *Bartonella* was identified by exploration of the genetic locus encoding a 17 kDa protein that was previously recognized as an immunodominant antigen of *B. henselae* (Padmalayam *et al*., 2000; Schmiederer and Anderson, 2000). The 17 kDa protein was shown to represent a VirB5-like protein of a T4SS that comprises the full set of essential homologues found in VirB-like systems (VirB2–VirB11), including the functionally associated T4CP VirD4 (Fig. 2). The genetic organization and sequence of the locus encoding the VirB/VirD4 T4SS is highly conserved in the available genome sequences of modern bartonellae, i.e. in *B. henselae*, *B. quintana* and *B. tribocorum* (Saenz *et al*., 2007). In the *B. tribocorum*–rat infection model, deletion mutants in *virB4* and *virD4* failed to get bacteraemic, thus demonstrating an essential role for the VirB/VirD4 system in establishing intraerythrocytic infection (Schulein and Dehio, 2002). Moreover, segregation analysis of complementation plasmids encoding either *virB4* or *virD4* in the respective mutant further demonstrated that the VirB/VirD4 system is required for colonization of the primary niche, but dispensable for the subsequent stage of intraerythrocytic infection (Schulein and Dehio, 2002). As the vascular endothelium is considered to be the main constituent of the primary niche (Dehio, 2005), endothelial cells may thus represent the primary target cells for the activity of the VirB/VirD4 system. Indeed, the VirB/VirD4 system mediates most of the cellular phenotypes associated with *B. henselae* infection of human umbilical vein endothelial cells (HUVECs) (Fig. 2). These include (i) massive rearrangements of the actin cytoskeleton, resulting in the formation and internalization of large bacterial aggregates by a unique structure known as the invasome, (ii) nuclear factor kappa B-dependent pro-inflammatory activation, leading to cell adhesion molecule expression and chemokine secretion, and (iii) inhibition of apoptotic cell death, resulting in enhanced endothelial cell survival (Schmid *et al*., 2004). These *in vitro* phenotypes are thought to relate both to the colonization of the primary niche in the reservoir host, and to the establishment of chronic infection of the human vasculature by *B. henselae* that may lead to vasoproliferation. Invasome-mediated internalization is considered to represent a specific mechanism for endothelial cell colonization *in vitro* (Dehio *et al*., 1997) that might reflect *in vivo* the characteristic bacterial aggregates found in bacillary angiomatosis lesions in association with proliferating endothelial cells (Dehio, 2003). The VirB/VirD4-dependent inhibition of apoptosis protects the cellular habitat of *Bartonella* in the primary niche and contributes indirectly to vasoproliferative growth due to an increased cell survival (Kirby and Nekorchuk, 2002). Finally, the VirB/VirD4-dependentpro-inflammatory activation of endothelial cells is thought to mediate the recruitment of phagocytes, which, upon activation by *B. henselae* infection, are triggered to release proangiogenic factors (such as vascular endothelial growth factor, VEGF) that promote endothelial cell proliferation in a paracrine manner (Kempf *et al*., 2001; Resto-Ruiz *et al*., 2002; Dehio, 2005). The direct mitogenic stimulation of endothelial cells as prominent phenotype of *B. henselae* infection turned out to be independent of the VirB/VirD4 system and was even found to be counterbalanced by VirB/VirD4-dependent cytostatic effects observed at high infection doses (Schmid *et al*., 2004).

Exploration of the genes encoded downstream of the *virB* and *virD4* loci in *B. henselae* has led to the identification of seven VirB/VirD4-translocated substrates, BepA–BepG (Bep = *B**artonella*effector protein) (Fig. 2) (Schulein *et al*., 2005). A deletion mutant for *bepA–bepG* is deficient in all VirB/VirD4-dependent cellular phenotypes of HUVECs, i.e. cell invasion, pro-inflammatory activation and the inhibition of apoptosis (Schulein *et al*., 2005). BepA–BepG thus represent the effectors that mediate VirB/VirD4-dependent cellular responses. The Beps display a modular structure. At the C-terminus, each effector contains a bipartite translocation signal composed of the approximately 140-amino-acid-large BID (Bep intracellular delivery) domain and a positively charged but unconserved tail sequence. Additional BID domains are present in a subset of the Beps (BepE–BepG), which apparently are not required for protein translocation via the VirB/VirD4 system, but rather have adopted effector functions within the host cell. Remarkably, BepG is composed exclusively of four BID domains. Other discernible domains are present in the N-termini of BepA–BepF. The paralogous proteins BepA, BepB and BepC carry a domain, FIC (filamentation induced by cAMP), which is present in many bacteria as well as in higher eukaryotes such as mammals. The molecular function of the FIC domain is unknown. BepD, BepE and BepF each contain tandem-repeated peptide motifs in their N-terminal regions that resemble eukaryotic tyrosine-phosphorylation motifs. Upon translocation into host cells, BepD was indeed shown to become tyrosine-phoshorylated (Schulein *et al*., 2005) and thereby potentially interferes with host cell signalling processes (Backert and Selbach, 2005). Tyrosine phosphorylation is also a feature of several effectors translocated by other bacterial pathogens, such as the T4SS effector CagA of *H. pylori*, or the T3SS effectors Tir and Tarp of enteropathogenic *Escherichia coli* and *Chlamydia trachomatis* respectively (Backert and Selbach, 2005).

The assessment of the molecular function of individual Beps and their contribution to the complex VirB/VirD4-dependent phenotypic changes of endothelial cells and potentially of other host cell types represent an interesting topic for future research. At present we are most advanced in our understanding of the molecular function of BepA. The capacity of *B. henselae* to inhibit the apoptosis of HUVECs was found to be entirely dependent on this effector protein. Upon translocation, BepA localizes to the plasma membrane, where it triggers the production of the second messenger cyclic AMP (cAMP) by a yet unknown mechanism. The resulting increase in the steady-state concentration of cAMP blocks effectively apoptotic cell death. The BID domain of BepA is sufficient to mediate membrane localization, elevation of cAMP levels and the resulting protection from apoptosis (Schmid *et al*., 2006). This domain, which evolved in bacteria as a part of a composite type IV secretion signal, has thus additionally acquired a function as targeting and effector domain within host cells.

## The Trw T4SS is required for erythrocyte infection

A second T4SS, Trw, was recognized in *B. henselae* in a search for promoters that are differentially regulated during infection (Seubert *et al*., 2003a). In the *B. tribocorum*–rat model, the Trw system is required for intraerythrocytic parasitism (Seubert *et al*., 2003a). Mutants deleted for the *virB10*-like *trwE* gene are deficient in establishing a long-lasting bacteraemia. However, as indicated by the short-termed appearance in blood on day five this mutant is probably still able to colonize the primary niche (i.e. the vascular endothelium) (Dehio, 2004; Schröder and Dehio, 2005). Thus, the Trw system appears to be dispensable for the infection of endothelial cells, but essential for the intracellular colonization of erythrocytes. Indeed, unlike wild-type *Bartonella birtlesii*, bacteria carrying mutations in the *trw* locus failed to cause bacteraemia in a mouse model (Mavris *et al*., 2005) as well as to invade murine erythrocytes *in vitro* (M. Vayssier, unpubl. results).

The Trw system of *Bartonella* represents a paradigm for a pathogenesis-related T4SS that evolved rather recently by functional diversification of a laterally acquired bacterial conjugation system (Frank *et al*., 2005; Nystedt *et al*., 2008). It displays characteristic features of a pathogenicity island and shares extensive similarity with the Trw conjugation system of the IncW broad-host range plasmid R388 (up to 80% amino acid sequence identity in individual components), which was originally isolated from different Enterobacteriacea. The *trw* genes of *Bartonella* are colinear with the respective genes of plasmid R388, except for the presence of multiple tandem gene duplications of *trwL* (homologous to *virB2*) and *trwJ–trwH* (homologous to *virB5–virB7*) (Fig. 2). Complementation of R388 derivatives carrying mutations in different *trw* genes with their *Bartonella* homologues allowed to demonstrate functional interchangability for some T4SS components (Seubert *et al*., 2003a; de Paz *et al*., 2005), underscoring the high degree of structural and functional conservation of individual subunits of these functionally diversified T4SSs. However, a major difference between the Trw systems is the lack of the T4CP-encoding gene *trwB* (homologous to *virD4*) in *Bartonella*. In R388, TrwB is required for export of the relaxase TrwC and the covalently attached DNA substrate. The absence of TrwB in *Bartonella* thus indicates that the *Bartonella* Trw system has lost its capacity to translocate substrates, unless the T4CP VirD4 of the VirB/VirD4 T4SS would promiscuously interact with the Trw system to replace the function of TrwB. It has been reported that the T4CP TraG of plasmid RP4 can functionally substitute TrwB for Trw-dependent plasmid mobilization in *E. coli* (Cabezon *et al*., 1994). However, no data are available supporting a functional interaction of the T4CP VirD4 and Trw in *Bartonella*.

The multiple copies of *trwL* and *trwJ* in the *Bartonella trw* locus encode variant forms of surface-exposed pilus components (homologues of VirB2 and VirB5 respectively). The other duplicated genes, *trwI* and *trwH*, encode homologues of VirB6 and VirB7, which are required for pilus elongation and for pilus anchorage to the outer membrane respectively. The presence of multiple copies of these T4SS components, which probably all are coexpressed, indicates that the primary function of the *Bartonella* Trw system may be the formation of variant pilus forms. Based on the essential role of the Trw system for erythrocyte invasion it is conceivable to assume that these variant pili may facilitate the interaction with different erythrocyte receptors, either within the reservoir host population (e.g. different blood group antigens), or among different reservoir hosts. Trw may thus represent a major determinant of host range/host specificity.

## Distribution of T4SSs in the genus *Bartonella* and their role in bacterial evolution

The genome sequence of *B. tribocorum* revealed the presence of a third T4SS in *Bartonella*, which is closely related to the VirB/VirD4 system and thus was termed Vbh (VirB*-*homologous) (Saenz *et al*., 2007). A systematic analysis of the distribution of the T4SSs VirB/VirD4, Vbh and Trw among all *Bartonella* species revealed important insights into the evolution of this pathogenic genus (Fig. 1). *B. bacilliformis* as representative of the ancestral lineage is the only *Bartonella* species that does not encode any T4SS, nor does its genome sequence reveal remnants of T4SS loci that would indicate their presence in an ancestor. All three T4SSs have thus been acquired by the modern lineage via lateral gene transfer. The lack of T4SSs in *B. bacilliformis* indicates that the common genus-specific infection strategy of the bartonellae (i.e. an infection cycle in endothelial cells and erythrocytes to cause a long-lasting intraerythrocytic infection) initially evolved in the absence of T4SSs. However, all species of the modern lineage appear to encode at least one T4SS that has adopted essential functions in this infection strategy, likely due to the replacement of virulence factors that are required for infection by *B. bacilliformis*. Moreover, as outlined in the following paragraphs, these T4SSs were crucial for the remarkable evolutionary success of the radially expanding modern lineage by their capacity to confer host adaptability.

Within the modern lineage, the Trw system is present in a large sub-branch comprising species that are adapted to diverse mammalian reservoir hosts, while it is absent from *Bartonella clarridgeiae* and the species of the ruminant-specific sub-branch, which diverted early during evolution of the modern lineage. The acquisition of Trw thus contributed to the capacity of modern bartonellae to adopt novel reservoir hosts within short evolutionary distances. Tandem gene duplication in combination with diversifying selection of the genes encoding pilus subunits may represent the molecular mechanism to adopt interaction with host-specific erythrocyte receptors as discussed above (Seubert *et al*., 2003b; Nystedt *et al*., 2008). Interestingly, the acquisition of Trw by the modern lineage correlates with the loss of flagella (Fig. 1), which are known to represent major pathogenicity factors for the invasion of erythrocytes by *B. bacilliformis* and probably other flagellated bartonellae (Dehio, 2004). In the evolution of the modern lineage, Trw may thus have functionally replaced flagella for mediating erythrocyte invasion.

The two other T4SSs present in species of the modern lineage, Vbh and VirB/VirD4, are closely related, and probably they are also functionally redundant. These VirB-like T4SS display an overlapping distribution – with each modern species encoding at least one of them. Vbh is present in the most deeply branching clade of the modern lineage. The lateral acquisition of the *vbh* locus by the modern lineage thus occurred coincidental with the separation from the ancestral lineage represented by *B. bacilliformis*. The phylogenetic origin of the Vbh system is unknown; however, it is closely related to the conjugation systems of some α-proteobacterial plasmids, such as of AvhB of plasmid pAT in *A. tumefaciens* (Saenz *et al*., 2007). Later in the evolution of the modern *Bartonella* lineage, the *virB/virD4* locus arose by duplication of *vbh*, or it was acquired by lateral gene transfer from a similar external source as *vbh*. Functional redundancy of the VirB-like T4SSs is suggested by the observation that Vbh appears to be functional only in species that do not encode VirB/VirD4 (e.g. as shown for *Bartonella schoenbuchensis*) (Saenz *et al*., 2007) (P. Engel and C. Dehio, unpubl. results). In contrast, Vbh apparently deteriorates in the presence of a VirB/VirD4 T4SS (ranging from the accumulation of mutations to an almost complete reduction of the *vbh* locus) (Saenz *et al*., 2007).

An integrated genomics approach in *Bartonella* allowed to infer an important role for VirB-like T4SSs in host adaptability as common characteristic of species in the modern lineage. VirB-like T4SSs are considered to facilitate bacterial adaptation to a given reservoir host – resulting in the typically low virulence potential of the modern species – as well as to novel hosts – resulting in radial speciation as signature for evolutionary success (Saenz *et al*., 2007). In contrast, the ancient *Bartonella* lineage represented by *B. bacilliformis* displays only a limited capacity for host adaptation, which resulted in the evolution of a single species that is highly virulent in its human reservoir. The molecular mechanism by which VirB-like T4SSs mediate host adaptability is probably dependent on their translocated effector proteins. Notably, the VirB/VirD4-translocated Beps show an atypically high degree of sequence variation among the closely related species *B. henselae* and *B. quintana*, suggesting an increased rate of evolution as the result of positive selection for adaptive functions in the infected host (Saenz *et al*., 2007).

## Conclusions

As exemplified by the ancestral pathogen *B. bacilliformis*, the common infection strategy of the bartonellae (i.e. colonization of endothelial cells and erythrocytes leading to a long-lasting intraerythrocytic infection) evolved initially without the contribution of T4SSs. However, within the radially expanding modern lineage, T4SSs have adopted diverse essential functions in this common infection process. These include the subversion of vascular endothelial cell functions by bacterial effectors proteins translocated by VirB-like T4SSs, and the interaction with erythrocytes by pilus-associated variant surface proteins expressed by the Trw T4SS. These T4SSs have probably facilitated the remarkable evolutionary success of the modern lineage by conferring host adaptability, which resulted in reduced virulence properties in a given host (providing a long-term fitness advantage for bacteria due to reduced damage of the infected host) and the adaptation of the generally host-restricted bartonellae to novel hosts. The molecular mechanisms facilitating T4SS-dependent host adaptability remain elusive; however, gene duplication and diversification by combinatorial sequence shuffling and point mutations seem to have contributed to the fast evolution of the translocated protein effectors of the VirB-like T4SSs (Saenz *et al*., 2007) and the surface-expressed pilus components of the Trw T4SS (Nystedt *et al*., 2008). Future research should focus on the elucidation of the molecular interactions of T4SSs and their effectors with host cells and the mechanisms of molecular evolution that govern host adaptability as novel role for T4SSs in bacterial pathogenesis.
